# Evaluation of Stream Mining Classifiers for Real-Time Clinical Decision Support System: A Case Study of Blood Glucose Prediction in Diabetes Therapy

**DOI:** 10.1155/2013/274193

**Published:** 2013-09-19

**Authors:** Simon Fong, Yang Zhang, Jinan Fiaidhi, Osama Mohammed, Sabah Mohammed

**Affiliations:** ^1^Department of Computer and Information Science, University of Macau, Macau, China; ^2^Department of Computer Science, Lakehead University, Thunder Bay, ON, Canada P7B 5E1

## Abstract

Earlier on, a conceptual design on the real-time clinical decision support system (rt-CDSS) with data stream mining was proposed and published. The new system is introduced that can analyze medical data streams and can make real-time prediction. This system is based on a stream mining algorithm called VFDT. The VFDT is extended with the capability of using pointers to allow the decision tree to remember the mapping relationship between leaf nodes and the history records. In this paper, which is a sequel to the rt-CDSS design, several popular machine learning algorithms are investigated for their suitability to be a candidate in the implementation of classifier at the rt-CDSS. A classifier essentially needs to accurately map the events inputted to the system into one of the several predefined classes of assessments, such that the rt-CDSS can follow up with the prescribed remedies being recommended to the clinicians. For a real-time system like rt-CDSS, the major technological challenges lie in the capability of the classifier to process, analyze and classify the dynamic input data, quickly and upmost reliably. An experimental comparison is conducted. This paper contributes to the insight of choosing and embedding a stream mining classifier into rt-CDSS with a case study of diabetes therapy.

## 1. Introduction

Clinical decision support system (CDSS) is a computer tool which broadly covers autonomous or semiautonomous tasks ranging amang symptoms diagnosis, analysis, classification, and computer-aided reasoning on choosing some appropriate medical care or treatment. Quoting from [1], a CDSS can be defined as “a system that is designed to be a direct aid to clinical decision-making in which the characteristics of an individual patient are matched to a computerized clinical knowledge base, and patient-specific assessments or recommendations are then presented to the clinician(s) and/or the patient for a decision.” As concise as this description goes, the brain of a CDSS is an automatic classifier which usually is a mathematically induced logic model. The model should be capable of mapping the relations between input events (usually are medical symptoms) and some predefined verdicts in the forms of medical advices/treatments. In other words, the classifier is delegated to predict or infer what the medical consequence will be, given the emerging events (sometimes medical interventions or prescriptions) as well as historic data that have been collected over time and induced into a classification model. The suggested medical consequences or so-called assessments and advices by the CDSS would be objectively recommended to a doctor for subsequent actions.

The underlying logics associated at the classifier of a CDSS are captures of knowledge or understanding between some attribute variables and the conclusion classes. The logics are represented either as some nonlinear mappings like numeric weights in an artificial neural network (black-box approach) or in some predicate-logic like IF-THEN-ELSE rules [2] known as clinical pathways. Traditionally the underlying logics are derived from a population of historic medical records, hence the induced model is generalized, versus which an individual new record can be tested for decision. The historic data are accumulated over time into a sizable volume for training the classification model. The records usually are digitized in electronic format and organized in a database [3]. Every time when a new instance of record is added, the classifier however needs to be rebuilt, in order to refresh its underlying logics to include the recognition of the new record. This learning approach is called “batch-mode” which inherits from the old design of many machine learning algorithms like greedy-search or partition-based decision tree: a model is trained by loading in the full set of data, and the decision tree is built by iteratively partitioning the whole data into hierarchical levels via some induction criteria. The short-comings of batch-mode learning have been studied and reported in [4], specifically its time latency in rebuilding the classification model whenever an additional record arrives.

The batch-mode learning kind of classifiers may work well with most of the CDSS when the updates over the ever-increasing volume of the medical records can be set periodic, and no urgency of a CDSS output is assumed. For example, the update for the CDSS classifier can happen at midnight when the workload of the computing environment is relatively low, and allowing for delay in inclusion of the latest records over 24 hours is acceptable for its use prior to the update. Most of the CDSS designs function according to this batch-mode approach (more details in Section 2) for nonemergency and perhaps nontime-critical decision-support applications, such as consultation by a general practitioner, nutrient advisor, and nursing care [5]. In general, CDSSs that adopt the batch-model learning while adequately meet the usage demands are those characterized by data that do not contain many fast-paced episodes and usually do not carry severe impacts. So there is little difference in its efficacy regardless the very latest records which are included in the training of the classifier or not. Examples are those decision applications over the data that evolve relatively slowly, which include but are not limited to common diseases that largely affect the world's population, cancers of which their treatments and damages may take months to years along the clinical timespan to take effect. In these cases, traditional CDSS with batch mode learning suffice their roles.

In contrast, a new type of CDSS called real-time clinical decision support system (rt-CDSS), as its name suggests, is able to analyze fast-changing medical data streams and can predict in real-time based on the very latest input events. Examples of fast-changing medical data are live feeds of vital biosignals from monitoring machines, like EEG, ECG, and EMG, as well as respiratory rate and blood oxygen level which are prone to change drastically in minutes or seconds. rt-CDSS usually is dealing with critical medical conditions, such as ICU, surgery, A&E, or mobile onsite rescue, where a medical practitioner opts for immediate decision-support by the rt-CDSS instrument based only on the latest measurements of his vital conditions. The information of vital conditions of the patient evolves very quickly during the course of operation, and it does matter of course in life and death.

As forementioned, a classifier is central to the design of CDSS, and the traditional batch-mode learning method obviously runs short for supporting a real-time CDSS due to its model refresh latency. As it was already pointed out in [6] the latency would increase probably exponentially as the training data size grows to certain amount; it means the classifier will become increasingly slow as fresh data continue to stream in, because of the continually training. In order to tackle with the drawback of batch-mode learning, a new breed of data mining algorithms called data stream mining has been recently invented [7] whose algorithms are founded on incremental learning. In a nutshell, incremental learning is able to process potentially infinite amount of data very quickly; the model update is incremental such that the underlying logics are refreshed reactively on the fly upon new instances, without the need of scanning through the whole dataset that embraces the new data repeatedly.

**Figure 1:**
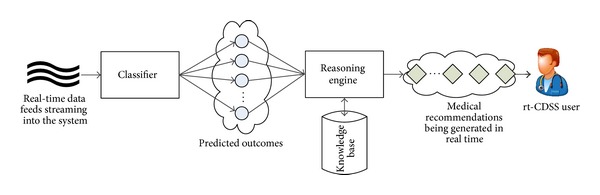
Block diagram of a general rt-CDSS system.

In the advent of incremental learning, new classifiers started to bring impacts into the biomedical research community. Some unprecedented real-time CDSS designs are made possible, in commercial prototype [8, 9] and in academic research [10–12]; even the developments are still in progress. These designs are characterized by having a real-time reasoning engine that is able to respond with fast and accuracy to clinical recommendation. The real-time decision generated by rt-CDSS is actually interpreted as a computer-inferred prediction from the given current condition of the patient that leads to further reasoning with an aid of a knowledge base, rather than a final decision confirmed by some authoritative human user. Generally there are two phases in the design of rt-CDSS, as shown in Figure 1. 

Live data feeds deliver real-time events to the classifier which learns the new data incrementally and be able to map the current situation to one of the predefined class labels as predicted outcomes. The predicted outcomes by the classifier are subsequently passed the reasoning engine that connects to a knowledge base for generating medical advices in real time, usually event driven. The reasoning engine could be implemented in various ways such as case-based reasoning or a novel approach [10] that embedded pointers at the decision tree leaves of the classifier, leading to some predefined guidelines of medical cure.

The focus of this paper however is on the real-time classifier, while the reasoning part of the rt-CDSS has already been discussed in [10]. The prediction by the real-time classifier here in the medical context is defined as a quantitatively guessed outcome that is likely to happen in the near future given the information of the current condition and the recent condition of the patients as well as the drug intake or clinical intervention, if any. Based on the predicted outcome, the rt-CDSS fetches the best option of cure correspondingly from a given knowledge base.

In our previous paper, we proposed a framework of rt-CDSS [10]; Very Fast Decision Tree (VFDT) was adopted as a candidate of a real-time classifier in the system design, because VFDT is classical and the most original type of stream-based classifiers [11]. Successively there are other variants modified from VFDT. Although VFDT is believed to be able to fulfill the role of real-time classifier in rt-CDSS, at least theoretically and conceptually, the performance has not been validated yet. As real-time classifier is the core of rt-CDSS, its performance must be able to fulfill the stringent criteria such as very short latency, very high accuracy, and very high consistency/reliability. This paper contributes to the insight of selecting and embedding a stream mining classifier into rt-CDSS with a case study of diabetes therapy that represents a typical real-time decision-making application scenario.

As a case study for comparative evaluation of classifiers for rt-CDSS, a computer-aided therapy for insulin-dependent diabetes mellitus patients is chosen to simulate a real-time decision making process in a scenario of dynamic events. The blood glucose level of diabetes patients often needs to be closely monitored, and it remains as an open question on how much the right dosage of insulin and the frequency of the doses should be given to maintain an appropriate level of blood glucose. This depends on many variables including the patient's body, lifestyle, food intake, and, of course, the variety of insulin doses. Along with this causal relationship between the predicted blood glucose levels and many contributing factors, multiple episodes can happen that may lead to different outcomes at any time. This is pertinent for testing the responsiveness and accuracy of the stream classifier considering that the episodes are the input values which may spontaneously evolve over time; the prediction is the guess work of the outcome based on the recent episodes.

The objective of this paper is twofold. We want to find out the most suitable classifier for rt-CDSS, and therefore we compared them in a diabetes therapy scenario. Also we want to test the performance of the classifier candidate all-rounded with a real-time case study, as a preliminary step to validate the efficacy of the rt-CDSS as a whole. Hence the study reported in this paper could serve as a future pathway for real-time CDSS implementation. The rest of the paper is structured as follow. An overview of classifiers that are used in CDSS is introduced in Section 2. The experiment to be conducted is described in details in Section 3. The second phase of rt-CDSS namely the decision inference is given in Section 4.  Section 5 concludes the paper.

## 2. Related Work

In the literature there are quite a number of clinical decision support systems being proposed for different uses. It is cautious that the type of the classifier has a direct effect on the real-time ability of CDSS. In this section, some related work on different medical applications is reviewed with the aim of pointing out the shortcomings of some legacy research approaches pertaining to rt-CDSS.

A recent report [13] discusses the potential of CDSS technology in breast cancer excerpted from multidisciplinary team meetings, as a synergy, by the National Health Service (NHS), in the United Kingdom. The report essentially highlighted the importance of CDSS in structural and administrative aspects of cancer MDTs such as preparation, data collection, presentation, and consistent documentation of decisions. But at an advanced level, the services of a CDSS should exceed beyond the use of clinical databases and electronic patients' records (EPRs), by actively supporting patient-centred, evidence-based decision-making. In particular, a beta CDSS called multidisciplinary team assistant and treatments elector MATE, is being developed and trialed at the London Royal Free hospital. MATE is equipped with functionalities of prognostication tools, decision panel where system recommendations and eligible clinical trials are highlighted in colors, and the evidential justification for each recommended option.

In the report, it was stated like a wish list that an advanced CDSS is able to evaluate all available patient data in real time, including comorbidities, and offer prompts, reminders, and suggestions for management in a transparent way. The purpose of the report is to motivate further research along the direction of advanced CDSS. Although it is unclear about which classifier that is built into MATE, incremental type of classifier would well be useful if it were to receive and analyze real-time data streams with very quick responsiveness.

On the other hand, a classical algorithm, namely, artificial neural network (ANN) has been widely used in CDSS. ANNs apply complex nonlinear functions to pattern recognition problems and generally yield good results. Szkoła et al. [14] built CDSS for laryngopathies by extending ANN algorithms that are based on the speech signal analysis to recurrent neural networks (RNNs). RNNs can be used for pattern recognition in time series data due to their ability of memorizing some information from the past. The data that the system deals with are speech signals of patients. Speeches are usually spoken intermittently, and they are hardly continuous data streams. In their case, rt-CDSS might not be applicable. The other group, led by Walsh et al, proposed an ensemble of neural networks for building a CDSS [15] for bronchiolitis for infants and toddlers. They showed that using an ensemble that works like a selection committee usually outperforms single neural networks.

There is another common type of conventional classification algorithms based on decision rules, for deciding how an unseen new instance is to be mapped to a class. Gerald et al. developed a logistic regression model showing those variables that are most likely to predict a positive tuberculin skin test in contacts of tuberculosis cases. Their paper [16] shows that a decision tree is developed into a CDSS for assisting public health workers in determining which contacts are most likely to have a positive tuberculin skin test. The decision tree model is built by aggregating 292 consecutive cases and their 2,941 contacts seen by the Alabama Department of Public Health over a period of 10 months in 1998. 

Another similar decision-support system called MYCIN [17] embeds decision rules into an expert system that provides interactive consultation. The decision rules are built into a simple inference engine, with a knowledge base of approximately 600 rules. MYCIN provided a list of possible culprit bacteria ranked from high to low based on the probability of each diagnosis, its confidence in each diagnosis' probability, the reasoning behind, and its recommended course of drug treatment. In spite of MYCIN's success, there is a debate about its classifier which essentially is an ad hoc sparked off. The rules in MYCIN are established on an uncertainty framework called “certainty factors.” However, some users are skeptical about its performance for it could be affected by perturbations in the uncertainty metrics associated with individual rules, suggesting that the power in the system was coupled more to its knowledge representation and reasoning scheme than to the details of its numerical uncertainty model [18]. Classical Bayesian statistics should have been used as suggested by some doubters. 

Iliad [19] which is a medical expert system software implementing Bayesian network as classifier has been developed by the University of Utah, School of Medicines, Department of Medical Informatics. In Iliad the posterior probabilities of various diagnoses are calculated by Bayesian reasoning. It was designed mainly for diagnosis in internal medicine. Currently it was used mainly as a classroom teaching tool for medicate students. Its power especially the Bayesian network classifier has not been leveraged for stream-based rt-CDSS.

Of all the well-known CDSS reviewed so far above, there is no suggestion indicating that they are operating on real-time live data feed; the data that they work on are largely EPRs, both patient-specific and of propensity, and perhaps coupled with clinical laboratory tests. Nevertheless, architectures of rt-CDSS namely, BioStream, [20], Aurora [21], and other monitoring devices [22] have been proposed which are specifically designed for handling medical data streams. 

BioStream, by HP Laboratories Cambridge, is a real-time, operator-based software solution for managing physiological sensor streams. It is built on top of a general purpose stream processing software architecture. The system processes data using plug-in analysis components that can be easily composed into any configuration for different medical domains. Aurora, by MIT, however is claimed to be a new system for managing data streams and for monitoring applications. The new element is the part of the software system that processes and reacts to continual inputs from many data sources of monitoring sensors. Essentially Aurora is a new database management system designed with a data model and system architecture that embraces a detailed set of stream-oriented operators.

From the literature review, it is apparent that research endeavor has been geared towards the direction of analyzing stream data, tapping the benefits of processing the physiological signals in real-time, and architecting framework of real-time stream-based software system. In 2012, Lin in his book chapter [11] discussed the state of the art and modern research trends of rt-CDSS; specifically he proposed a web-based rt-CDSS with a full architecture showing all the model-view-controller components. In-depth discussions are reported from process scheduling, system integration, to a full networked infrastructure. It is therefore evident that real-time decision system is drawing attentions from both industry and academia, although the details of the analyzer component is still lacking. In [10] we advocated that the main piece of an effective rt-CDSS is an incremental learning model. By far there is no study dedicated to investigate the classifiers for handling data streams in rt-CDSS, to the best of the authors' knowledge. This paper is intended to fill this missing piece.

## 3. Predicting Future Cases: Problem Definition

As a case study of evaluating the performance of several types of classifiers to be used in rt-CDSS, a diabetes therapy is used. The basis of the diabetes therapy is to replace the lack of insulin by regular exogenous insulin infusion with a right dosage each time, for keeping the patients alive. However, maintaining the blood glucose levels in check via exogenous insulin injection is a tricky and challenging task. Despite the fact that the reactions of human bodies to exogenous insulin vary, the concentration of blood glucose can potentially be influenced by many variables too [1]. These variables include but are not limited to, BMI, mental conditions, hormonal secretion, physical well-being, diets, and lifestyles. Their effects make a synthetic glucose regulation process in diabetic patients highly complex as the bodily reaction to insulin and other factors differs from one person to another. It is all about a matter of a right dosage and the right timing of insulin administration, for regulating the fluctuation of blood glucose concentration at a constant level. Hyperglycemia can occur when the blood glucose level stays chronic above 125 mg/dL over a prolonged period of time. The damages are on different parts of the body, such as stroke, heart attack, erectile dysfunction, blurred vision, and skin infections, just to name a few. At the other end, hypoglycemia occurs when the content of glucose ever falls below 72 mg/dL. Even for a short period of time, hypoglycemia can develop into unpleasant sensations like dysphoria and dizziness and sometimes life-threatening situations like coma, seizures, brain damage, or even death. The challenge now is to try to adopt a classifier which incrementally learns the pattern of a patient's insulin intakes and predicts his blood glucose level in the near future. Should there be any predicted outcome that falls beyond the normal ranges, the rt-CDSS should give a remedy recommendation.

### 3.1. Data Description

The data used in this experiment are the empirical dataset from AAAI Spring Symposium on Interpreting Clinical Data (http://www.aaai.org/Press/Reports/Symposia/Spring/ss-94-01.php). This data represents a typical flow of measurement records that would be found in any insulin therapy management. The live data feed can serve as an input source for rt-CDSS for the sake of forecasting the condition of the patient in the near future as well as offering medical advice if necessary. The insulin-dependent diabetes mellitus (IDDM) data are event-oriented data because the data is a temporal series of events. Typically there are three groups of events in an insulin therapy, blood glucose measurement (both before/after meals and ad hoc), insulin injections (of different types), and amount of physical exercises. The events are time stamped. However, there is no rigid regularity on how often each of these events would happen. A rough cyclical pattern can be however observed that goes by spacing the insulin injections, probably several times over a day, and the corresponding cycle of blood glucose fluctuation follows closely. These cycles loop over day after day, without specifying the exact timing of each event. One can approximately observe that an average of three or four injections are being applied.

**Figure 2:**
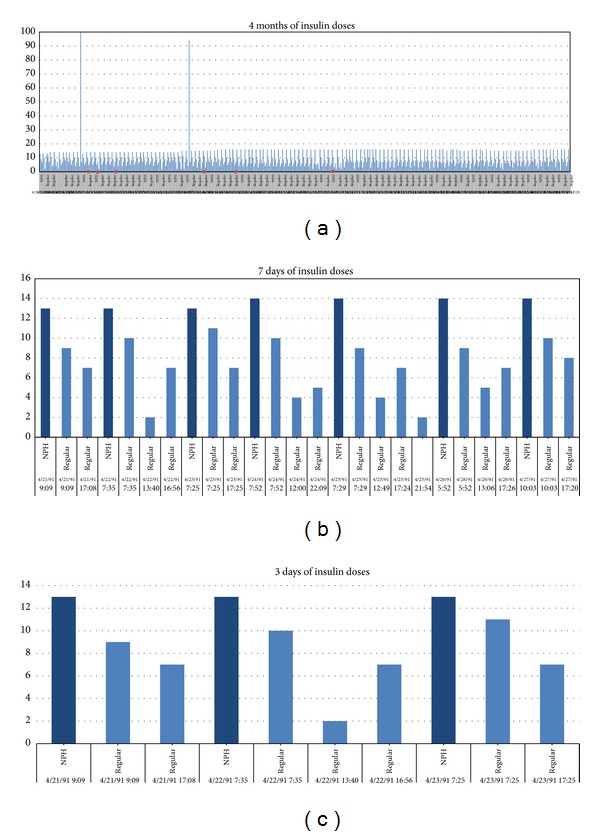
Periodic patterns of IDDM events, data taken from a subset of AAAI Spring Symposium on Interpreting Clinical Data. (a) Adaption of insulin for 4 months. (b) Adaption of insulin injections for 7 days. (c) Adaption of insulin injections for 3 days.

**Figure 3:**
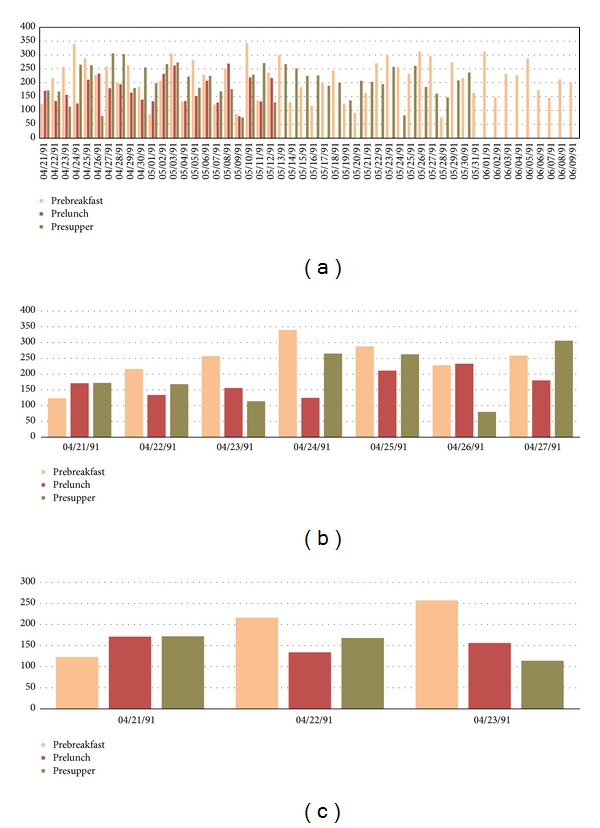
Periodic patterns of blood glucose measurements, data taken from a subset of AAAI Spring Symposium on Interpreting Clinical Data. (a) Time scale of 50 days. (b) Time scale of 7 days. (c) Time scale of 3 days.

In Figure 2, a sample of these repetitive cycles of events is shown for illustrating the synchronized events. Events of insulin injections and blood glucose measurements are more or less interleaved loosely periodically over time; exercises and sometimes hypoglycemia occur occasionally. In the example presented in Figure 2, two views are provided. The 4-months adaption of insulin injection shows a relatively long-term pattern over time (Figure 2(a)); two exceptionally high doses of insulin over units of 100 were given; more importantly the insulin pattern is never periodically exact, although some cycles are seen to be repeated [23]. The overall insulin intake looks increasing over time from the initial month to the last month. Some events of hypoglycemia have occurred too, sporadically, as represented by red dots in the graph. Zoomed-in views are shown in Figures 2(b) and 2(c), where the timing of the insulin injections are clearly seen. Though the insulin injections are repeating over time, the exact times of injections are seldom the same for any two injections. Sometimes, neutral protamine Hagedorn (NPH) and regular types of injections are taken at the same time.  Figure 3(a) shows a change of habit in blood glucose measurements; the frequency has reduced across fifty days by dropping the prelunch and presupper measurements. Figures 3(b) and 3(c) show the same but in time scales of 7 days and 3 days, respectively. The graphs demonstrate a fact that the patterns of timing and doses of insulin injections are aperiodic that elicits substantial computational challenges in testing the classifiers.

### 3.2. Prediction Assumptions

In order to engineer an effective real-time clinical decision support system, we should use a classification algorithm that can analyse data efficiently and accurately. Traditional decision tree may be a good choice; however, it cannot handle continuous rapid data. To alleviate this problem, incremental classification algorithm, such as VFDT, should be used. For easy illustration when it comes to describing the system processes and workflows throughout this paper, the term VFDT is used that generalized the category of incremental learning methods. In fact, however, other algorithms can be exchanged. Different incremental classifiers in the rt-CDSS model can be adopted. 

**Figure 4:**
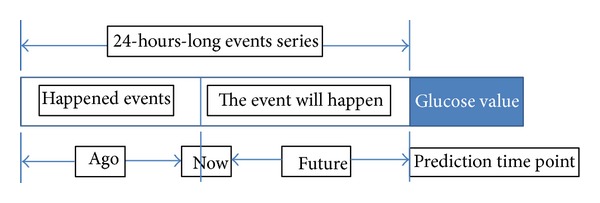
Sliding window for incremental classifier.

The prediction is rolling as time passes by. The initial model construction takes about a small portion of the initial data after which the classifier learns and predicts at the same time. One can imagine that there is a time window of 24 hours; when new data rolls in, the old data are flushed out from the memory of the classifier. This way, the classifier can be adaptive to the most current situation and will keep its effectiveness in real time all the time. Regardless of the total size of the data which potentially amount to infinity, the rt-CDSS which is empowered by the incremental learning classifier will still work fine. So in our design, a changing period of 24 hours would be covered for both events that have already happened and will likely happen. Within this period, the classifier continually analyses and remembers the causal relationship between the happened events and the future events. As a case study, the classifier is made to predict future blood glucose level, given the events of insulin injections, meals, and historical blood glucose levels as they all carry certain effects predicting future blood glucose level. The concept of the sliding time window is shown in Figure 4.

As we know, a blood glucose measurement is taken; the measured value is affected by a composite of events that happened during the last several hours. The event may be a meal, an exercise, or an insulin injection. In the design of our experiment, we consider the events which happened during the last 24 hours before the last prediction time point. There are 3 kinds of insulin injections given in the dataset, they are regular insulin, NPH insulin and Ultralente insulin. Regular insulin has at most 6 hours duration effect, NPH has at most 14 hours duration effect, and Ultralente insulin has 24 hours duration effect. Once the prediction point is passed, another fresh set of 24-hours-long events series (24 hours before the previous prediction time point) is loaded to the classifier. This event series include two parts, one is happened event; this part will be extracted from the collected data feed from the monitoring device of the system. For example, assume now that the time is 10:00 we want to predict the blood glucose level at 17:00. Then the system will extract the events data list from yesterday 19:00 to today 10:00 (now), and from the averaged historic record patterns we infer what events the patient would most like to part take in the next 7 hours (from 10:00 to 17:00), such as lunch, snack and exercise. This is to emulate the lifestyle pattern taking into consideration the causality relation between two consecutive days. Some events like meal, exercise, and regular insulin injection only have short effect duration; for these events we only consider the case in the past 6 hours or 3 hours depending on the effect duration of the insulin.

### 3.3. Event List

The data source where the diabetes time-series dataset to be used for our experiment is UCI archive (http://www.ics.uci.edu/~mlearn/) which is popular for benchmarking machine learning algorithms. The events in the diabetes dataset are indexed by numeric codes. Totally there are 20 codes in code list, but not every code is relevant to the blood glucose level which is our predicted target. Some codes are measurements they can provide a blood glucose value and they also represent an event. For example, code 58 represents the event of prebreakfast that means it will happen soon, and it gives the blood glucose count before the breakfast. Code 65 is hypoglycemia symptom that is being measured. The event occurs whenever a measurement of hypoglycemia is detected positive. And there are many different codes that may refer to the same event, such as code 57 and code 48. So we need to simplify the code list and retain only valid events in this list.

**Figure 5:**
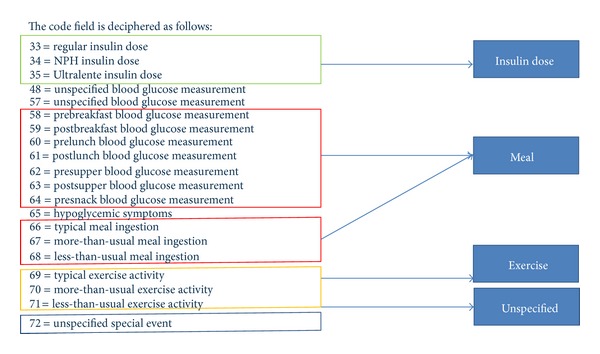
An event list describing the events by codes.

From Figure 5 we can see that only four events have effects on the blood glucose levels. The event meal includes several codes, some of them represent a measurement before or after a meal we consider them also representing the time of a meal. For example, when code 58 (with value 100) appears at 9:00, we can know that this person will eat breakfast at nearly 9:00, and the blood glucose before his breakfast is 100. So after simplifying the code list, 4 valid events remain. Each event may have several types. For instance, the event insulin dose has 3 types: regular insulin, NPH insulin, and Ultralente insulin. Below is a short list of various types shared by the events.

- Event insulin dose: regular insulin, NPH insulin, and Ultralente insulin.
- Event meal: breakfast, lunch, supper, snack, typical meal, more than usual, less than usual.
- Event exercise: typical, more than usual, less than usual.
- Event unspecified special event: exist and N/A.

### 3.4. The Structure of Training/Testing Instance

All classifiers work on multivariate data which is formatted as an instance of multi-attributed record *x*_*i*_ and it must be described by a set of features (*a*_1_,*a*_2_,…,*a*_*m*_)_*i*_ and a corresponding class label *y*_*i*_. In this case of diabetes therapy, the data are in time series. A preprocessing software is programmed to convert the events over a time frame of 24 hours into a multiattributed records of *m* dimensions in *n* rows (instances).

**Figure 6:**
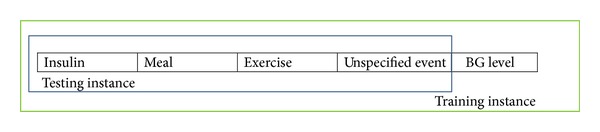
Data instance structure for training/testing a classifier.

As described in Section 3.3, the events are filtered so only the relevant event types are used to compose the instances for training and testing. The structure is shown in Figure 6. 

According to the general structure specified in Figure 6, a total of 16 attributes would be computed from the event list as follow:

 
*A*0: measurement code
 
*A*1: how long ago regular dose
 
*A*2: how much regular dose
 
*A*3: how long ago NPH dose
 
*A*4: how much NPH dose
 
*A*5: how long ago Ultralente insulin dose
 
*A*6: how much Ultralente insulin dose
 
*A*7: the unspecified event in past 6 hours
 
*A*8: blood glucose level for the previous 3 days
 
*A*9: hypoglycemia in the past 24 hours
 
*A*10: last meal in past 6 hours
 
*A*11: how long ago the last meal in the past 3 hours
 
*A*12: how long ago the last exercise in the past 24 hours
 
*A*13: how much exercise
 
*A*14: Patient ID
 
*A*15: Blood glucose level (just for training instance).

*A*8 is the reference blood glucose level (BGL), which is very important for future blood glucose level prediction. It depends on the BGL in the previous 3 days. From the data analysis we found that there is an important relationship between the current BGL and historical blood glucose level, that exists in the same time period during the previous three days. And we found that the BGL of just one day ago has the most important effect, we call it the factor “1 day before,” “2 days before” has second most important effect, and last is “3 days before.” So weights of relative importance are arbitrarily set for the 3 factors and *w*_1_ = 0.5, *w*_2_ = 0.3, *w*_3_ = 0.2. *F*_1_ = 1 day before, *F*_2_ = 2 days before, and *F*_3_ = 3 days before. The simple formula that generates the reference BGL, *R*, is *R* = ∑_*i*=1_^3^ *F*_*i*_ *W*_*i*_ where *f* is the factor and *w* is the weight.

### 3.5. Target Classes

The target class is the prediction result about blood glucose level. Instead of predicting a precise numeric value, the classifier tries to map a new testing instance to one of the 7 classes that describes basically whether the BGL is normal or not.  Table 1 shows a class table that illustrates the seven possible normal/abnormal blood glucose levels and their meanings.

As we all know that the blood glucose level will rise up after meals, and it will return to normal level after about 3 hours. So we need to consider the event meal in only the past 3 hours when we do the prediction. In normal situation, one hour postprandial BGL is ranging from 120 to 200 mg/dL (Normal_1) and 2 hours postprandial BG level is ranging from 70 to 140 mg/dL (Normal_2).

## 4. Experiment

### 4.1. Experimental Environment and Design

The software system prototype of the rt-CDSS including the classifier is built by Java programming language. The system makes external application-interface calls to the classification algorithms provided by Massive Online Analysis (MOA) (http://moa.cms.waikato.ac.nz). The operating system is MS-Windows 7, 64 bits edition, and the processor is Intel i7 2670 QM 2.20 GHz. 

There are 70 diabetes records in our dataset that are collected from 70 different real patients. Each record covers several weeks' to months' diabetes data. We divide every record into two parts; one represents the historical medical data for training and the other part represents future medical data for testing. We use the first part to train the system with incremental classification algorithms, and we use the second part to do the accuracy test. In reality, when using the system to do a prediction for a new patient, the patient's historical medical record would be loaded in beforehand for initial boot-up training. The historical medical record can be of length of several days (or weeks) of diabetes events. In our experiment, we save the first 1% records from each record as the boot-up training data set.

Firstly, we will conduct the accuracy test for VFDT, iOVFDT [24], Bayes [25], and Perceptron (which is a classical implementation of ANN) [26], respectively. Default parameters are assumed. Then we will analyses their accuracy performance and from there we choose the qualified algorithms for further consistency testes. Finally, we will determine which algorithms work best in our rt-CDSS environment.

### 4.2. Accuracy Test

All the 70 original patients' records that are available from the dataset would be used for the accuracy test. There are 70 independent accuracy tests. Every record is tested individually using the candidate classifiers and their accuracies are measured, by considering the past 24 hours window of data as training instances, and the testing starts from the first day of the data monitoring till the last. The 70 records are run in sequential manner for the classifiers. Since each instance carries a predefined BGL label, after running through the full course of prediction, the predicted results could be compared with the actual results. By definition, the accuracy is given as accuracy = (total number of correctly classified instances/the total number of instances available for this particular patient) × 100. The total accuracy is therefore the average of the accuracies over 70 patients' BGL predictions during the course of diabetes therapy. The overall statistics of the accuracy tests are shown in Table 2.

From Table 2, it is observed that the average accuracy for all the candidate algorithms are acceptable except Perceptron. For the algorithms that have acceptable accuracies such as VFDT, iOVFDT, and Bayes, over 75% of the cases they are predicting are at an accuracy higher than or equal to 81%. That means in most situations the rt-CDSS with these qualified algorithms are making useful predictions. For Perceptron, however, during the prediction course of 75% of the records its accuracy is lower than 53.814%, that is just marginally better than random guesses. As a concluding remark, Perceptron fails to adequately predict streaming data when the initial training sample is just about 10%. Thus it is not a suitable candidate algorithm to be used in rt-CDSS when the incoming data stream is dynamic, complex, and irregular. 

**Figure 7:**
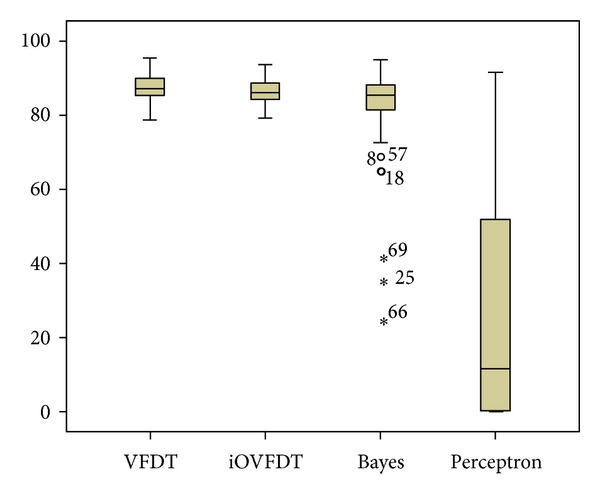
Boxplot diagram of accuracy performances for the classifiers.

Figure 7 is a boxplot diagram for comparing visually the performances of the candidate algorithms. Boxplot diagram is an important way to graphically depict groups of numerical data through their quartiles. It is often used as a method to show the quality of a dataset, where in this case the performance results of it.

From the boxplot, we can see that the performances between VFDT and iOVFDT are so close; their accuracy distributions are very similar, and there is no outlier in their distributions. The maximum accuracy for iOVFDT is slightly lower than that of VFDT, but iOVFDT has an overall consistent accuracy performance and a higher minimum accuracy compared to VFDT. That is because iOVFDT was designed to achieve optimal balance of performance, where the result may not be maximum but well balanced in consideration of the overall performance.

For Bayes algorithm the accuracy is basically acceptable, but there are 3 outliers. These extreme values are associated with records 69, 25, and 66, where the accuracies fall below 50%. It means Bayes works well for most of the records, but there also exist some situations where Bayes fails to predict accurately. The worst performance as seen from the boxplots is by Perceptron; in most cases, it predicts incorrectly.

**Figure 8:**
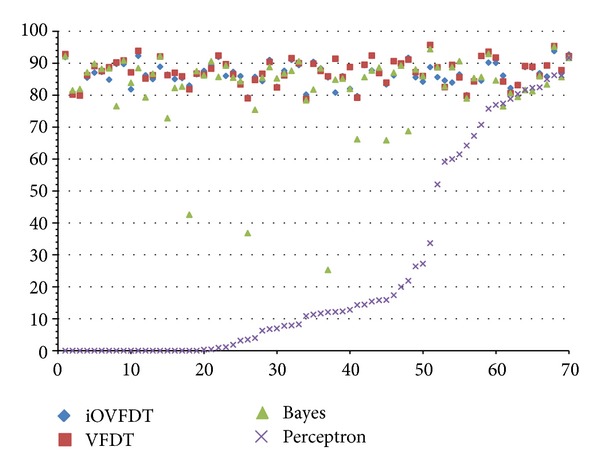
Scatterplot diagram of accuracy performances for the classifiers.

The scatter plot as depicted in Figure 8 shows an interesting phenomenon when the accuracy results are viewed longitudinally across the whole course of prediction in rt-CDSS. The qualified classifiers such as VFDT, iOVFDT and Bayes are all able to start showing early high accuracies especially for VFDT and iOVFDT. They are able to maintain this high level of accuracies across the full course at over >80%. The performance for Bayes is also quite stable starting from the initial record to the end, except several outlier points.

In contrast, Perceptron picked up the accuracy rate after being trained with approximately 25 sets of patients' records; the accuracy trend increases gradually over the remaining records and climbs up high on par with the other classifiers near the end. In fact, its maximum accuracy rate is 91.667%, while the other prediction accuracies for the other classifiers range from 93.793% to 95.681%. And the accuracy for Perceptron algorithm seems to be able to further increase should the provision of training data be continued. This implies that Perceptron algorithm is capable of delivering good prediction accuracy, but under the condition that sufficient training data must be made available for inducing a stable model. However, in scenario of real-time data stream in which rt-CDSS is embracing, incremental learning algorithms have their edge in performance.

Overall, with respect to accuracy, the best performers are VFDT and iOVFDT. The performance for Bayes is acceptable though outliers occur at times. Given the fact that Perceptron is unable to achieve an acceptable level of accuracy in the initial stage of incremental learning, it is dropped from further tests in our rt-CDSS simulation experiment. The remaining qualified algorithms are then subject to further tests.

### 4.3. Consistency Test

Kappa statistics is used for testing the consistency of accuracies achieved by each of the VFDT, iOVFDT, and Bayes classifiers. Kappa statistics is generally used in data mining, statistical analysis, and even assessment of medical diagnostic tests [27], as an indicator on how “reliable” a trained model is. It basically reflects how consistent the evaluation results obtained from multiple interobservers are and how well they are agreed upon. A full description of the Kappa statistics can be found in [28]. Generally a Kappa of 0 indicates that agreement is equivalent to chance, where as a Kappa of 1 means perfect agreement. It loosely defines here as a measure of consistency by saying a model that has a high Kappa value is a consistent model that would expect about the same level of performance (in this case, accuracy) even when it is tested with datasets from other sources. The Kappa statistics is computed from the 70 patients' records via a 10-fold cross-validation with each fold of different combination of partitions (training and testing) as different inter-observers, randomly picked from the whole dataset.

The definition of Kappa statistic is defined as *K* = (*Po* − *Pc*)/(1 − *Pc*), where *Po* is the observed agreement and *Pc* is chance agreement. The results of the Kappa statistics from the candidate classifiers are tabulated in Table 3.

We can see from Table 3 that the Bayes classifier has the highest consistency value relatively; it belongs to the substantial group of Kappa statistics. The other 2 algorithms are located in the moderate group. The result shows that all the three algorithms have considerably moderate and substantial consistency in rt-CDSS. Higher Kappa statistics are yet to be obtained probably due to the irregularity of events in the datasets and of the 70 patients the diabetes therapy patterns vary a lot. 

### 4.4. Test of ROC Curve and AUC

ROC is an acronym for Receiver Operating Characteristic; it is an important means to evaluate the performance of a binary classifier system. It is created by plotting the fraction of true positives out of the positives (TP = true positive rate) in *x*-axis and the fraction of false positives out of the negatives (FP = false positive rate) in *y*-axis. The terms positive and negative describe the classifier's prediction results, and the terms true and false refer to whether the prediction results correspond to the fact or not. The standard contingency table or confusion matrix for binary classification is shown in Table 4.

In our rt-CDSS, the classifiers are multiclasses classifiers rather than binary classifiers as they predict the future conditions of the patients into one of the seven BGLs. So we need a modification to extend the conventional ROC curve in our evaluation experiment. By following the modification which was reported in [29], a binary ROC curve is extended for the use for multiclasses classifiers. The modified contingency table is shown in Table 5.

The occurrences for TP and FP for each class are counted respectively. A small assumption is made during the counting: when counting for a class, an instance in this class is counted as “yes” and the instances in other class are “no”, just like binary classifier. Then by adding up all the TPs as total TP_multi_ and all the FPs as total FP_multi_, we compute the TP_multi_ and FP_multi_ and derive a composite ROC by calculating sensitivity and specificity as the *y*-axis and *x*-axis of the ROC chart accordingly.

Sensitivity is named after TP_multi_ which is sometimes called recall rate. It counts about the proportion of actual positives which are identified correctly by the classifier. The proportion here is the percentage of diabetes patients of abnormal BGL who are correctly predicted as having the abnormal condition in the rt-CDSS. Specificity which is the FP_multi_ and sometimes known as the true negative rate measures the proportion of negatives which are predicted correctly as such. The proportion is the percentage of diabetes patients with normal BGL who are correctly predicted as not having the abnormal BGL.

**Figure 9:**
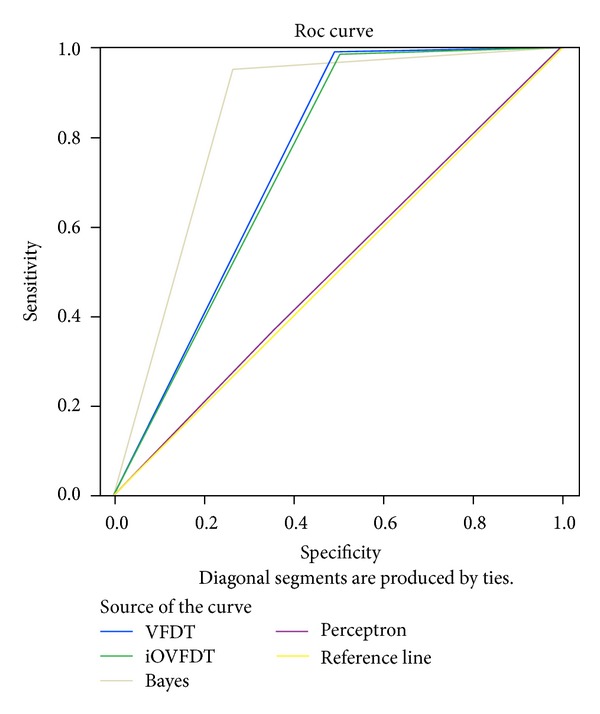
ROC and AUC performances of the classifiers.

Ideally a perfect classifier should be 100% sensitivity and 100% specificity, meaning it can predict that all patients who will have abnormal BGL really will have the condition; patients who will not have the abnormal BGL will actually be free from it. So when plotting sensitivity and specificity on a ROC plot, the curve should be the higher the better in these two directions. Theoretically any classifier will display certain trade-off between these two measures. For example, in rt-CDSS in which the user is testing for extra precaution for health assessment for the diabetes patient, the classifier may be set to consider more thorough life events that may be related to a sudden change in BGL, even though they are minor ones (low specificity), and perhaps higher influential factors are adjusted for these event variables that may directly or indirectly trigger the change in BGL (high sensitivity). This trade-off can be perceived graphically by the shape of the ROC. The ROCs for the classifiers are shown in Figure 9. The corresponding AUC numeric results of the ROCs are tabulated in Table 6.

From the ROC curve and AUC (area under the curve) as shown in Figure 9 and Table 6, we can see that the Bayes classifier has the largest AUC, and the larger an AUC is the better performance it gives. VFDT and iOVFDT have almost the same AUCs. It means their performances in the rt-CDSS model are very close. Perceptron has the smallest AUC in this design, amounts to nearly 0.5; it means that the classifier works almost randomly.

### 4.5. Test of Precision, Recall and F-Measure

In pattern recognition and data mining, precision is the fraction of relevantly retrieved instances. In the situation of rt-CDSS classifications, precision is a measure of the accuracy provided that a specific class has been predicted. It is calculated by this simple formula: precision = TP/(TP + FP). 

Recall is defined as the fraction of relevantly retrieved instances. We can infer that the same part of both precision and recall is relevance, based on which they all make a measurement. Usually, precision and recall scores are not discussed in isolation, and the relationship between them is inverse, indicating that one increases and the other decreases. Recall is defined as recall = TP/(TP + FN).

In a classification task, recall is a criterion of the classification ability of a prediction model to select labeled instances from training and testing datasets. A precision with score 1.0 means that every instance with label belonging to the specific class (predicted by the classifier) does indeed belong to that class in fact. Whereas a recall of score 1.0 means that each instance from that particular class is labeled to this class and all are predicted correctly, none shall be left out.

*F*-measure is the harmonic mean of precision and recall, that is: *F*  measure = 2/((1/Precision)+(1/Recall)) = (2 · Precision · Recall)/(Precision + Recall). It is also known as balanced *F* score or *F*-measure in tradition, because recall and precision are equally weighted. The general formula for *F*_*β*_ measure is *F*_*β*_ = (1 + *β* ^2^)/((1/Precision)+(*β* ^2^/Recall)) = ((1 + *β* ^2^) · Precision · Recall)/(*β* ^2^ · Precision + Recall). As mentioned before, precision and recall scores should be taken into account simultaneously because they have a strong relation essentially. Consequentially, both are combined into a single measure, which is *F*-measure, which is perceived as a well-rounded performance evaluation, more highly valued than the simple accuracy.

**Figure 10:**
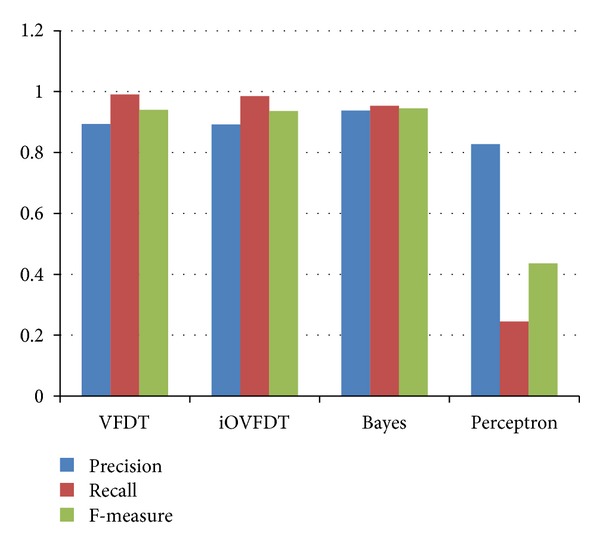
Performances of the classifiers in terms of precision, recall, and *F*-measure.

The performance results of precision, recall, and *F*-measure are then tabulated in Table 7 and shown in bar-chart in Figure 10.

With respect to precision value, we can see Bayes has the highest. The precision values between VFDT and iOVFDT are nearly identical. There is a strange observation that the precision score for Perceptron is also quite high (0.827), despite the fact that Perception was most down rated in the accuracy test. This phenomenon can be explained that Perceptron rarely makes a positive decision; from the histogram above in Figure 10 we can easily see that the total sums of TP and FP for perceptron is much less than the others. But out of these rare predictions, Perceptron has a relatively high rate in precision.

When it comes to recall criterion, VFDT has the best score, iOVFDT and Bayes both have good Recall values (>0.95). We can see quite clearly that Perceptron made a lot of false negative prediction, as its Recall value is only 0.245. This is an immature sign of its underlying model is under-trained with insufficient training samples.

**Figure 11:**
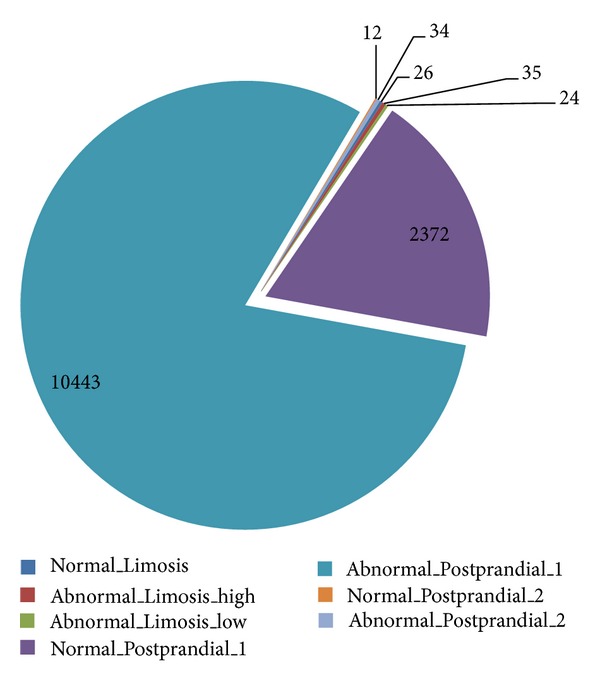
Distribution of the instances among the target classes.

For the final composite scores, *F*-measures, as shown in Table 7, Bayes outperforms the rest of the others. The candidate that has the second highest *F*-measure is VFDT whose difference is merely 0.005. In summary, Bayes classifier can be a good candidate for implementing rt-CDSS given the fact that it overall outperforms the rest in Precision, *F*-measure, AUC and Kappa statistics. However one drawback is the outlier predictions that can occur at Bayes classifier though seldom in the course of prediction. In medical applications, such anomaly in performance can lead to grave consequences. The underperformance of Bayes may be due to a large amount of conflicting conditions in the dataset where a particular class out of the seven classes is highly unbalanced (biased). As shown in Figure 11, the class called *Abnormal_Postprandial_1* has an unusually high number of instances (10,443) compared to the rest of the classes.

**Figure 12:**
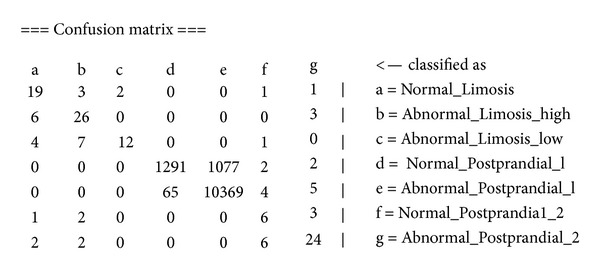
Confusion matrix of Bayes classifier.

**Figure 13:**
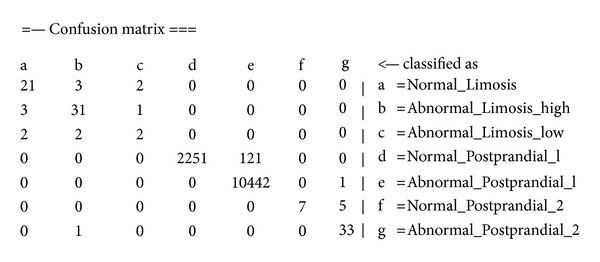
Confusion matrix of OVFDT classifier.

By comparing the confusion matrices of Bayes and OVFDT, as shown in Figures 12 and 13, respectively, one can observe the reason behind the shortcoming of Bayes prediction. In the biased class which dominates most of the training instances, Bayes incorrectly classified 1,077 instances pertaining to *Abnormal_Postprandial_1* compared to OVFDT which classified wrongly of 121 for the same class. This particular inaccuracy at the biased class rated down the performance of Bayes as a whole given its somewhat rigid probabilistic network. On the other hand, decision tree type of classifiers such as OVFDT and iOVFDT are about to grow extra decision paths to relieve this specific inaccuracy hotspot.

iOVFDT is the second best in Kappa statistics, and provides reasonably well performance in the other measures (though not the highest). It could be an appropriate choice in rt-CDSS given its stable performance considering all aspects of evaluation. Perception is unsuitable for classifying data stream.

## 5. Conclusion and Future Works

Clinical decision support system (CDSS) has drawn considerate attentions from researchers from information technology discipline as well as medical practitioners. This is a sequel paper which follows a new novel design of real-time clinical decision support system (rt-CDSS) with data stream mining. In our previous paper, a conceptual framework has been proposed. However, one important internal process which is the core of the whole system is the classifier which is supposed to predict the future condition of a patient based on his past historic events as well as other generalized medical propensity information. Once a prediction is made, the leaf pointers of the class nodes of the decision tree will fetch the relevant prescribed medical guidelines for recommendation. It can be understood that such classifier inside the rt-CDSS would need to possess the following capabilities: (1) handling data stream such as live feeds of biosignals monitoring devices, other instant measurements of vital signs, and physiological reactions/responses to drugs treatments; (2) a very short time delay in model updates when new data arrives; and perhaps most importantly (3) accurate and consistent prediction performance. 

Traditional classifiers which have been widely used in CDSS and whose designs based on structured electronic patients' records (instead of stream data) are known to come short of satisfying the three requirements. The main distinction between traditional CDSS and rt-CDSS is the reaction time required; CDSS is centered on disease that has certain length of onset time and rt-CDSS is for emergency medical situations; hence timely and accurate decisions are very crucial. It was already studied in the other papers that traditional classifiers require a complete scanning of a full training dataset every time a new piece of data is added on. Such batch-based learning is not efficient enough to learn and adapt to fast moving data stream in real-time. rt-CDSS is a new breed of decision support tools. To the best of the authors' knowledge, none of the related works has investigated the issue of finding a suitable classifier for rt-CDSS. This paper contributes to a performance evaluation of several incremental learning algorithms together with an artificial neural network algorithm that has been used extensively for traditional CDSS. A case study of diabetes therapy with real patents' data was used in the evaluation experiment which simulates a therapeutic decision-support scenario where real-time blood glucose level is predicted based on various insulin intakes and life-time events.

Our results show that classifier of artificial neural network gives unsatisfactory performance under a rolling sequence of event data. A neural network usually needs to be sufficiently trained by the full volume of dataset which may not be available in data streaming environment. Bayes algorithm is found to be having the highest consistency in terms of Kappa statistics and few other performance scores; its prediction is stained with some outliers (sudden accuracy degradations) in the course of prediction. VFDT on the other hand has the highest accuracy, but its accuracy for one dataset may not always be as consistent as that for another dataset, when compared to iOVFDT whose performance is rather stable.

As future works, the authors are inclined to test a wider range of stream mining algorithms that are available in the literature. The same performance testing would be repeated while the classifiers are to be integrated with the other components of the rt-CDSS and be tested as a whole system. Scenario and dataset of higher complexity should be tested with the classifiers too, for example, ICU data where multiple data feeds (ECG, respiratory measures, blood pressure, oxygen in blood, etc.) are streaming into the rt-CDSS in real time.

## Figures and Tables

**Table 1:** Seven possible target classes.

Target class	BG range (mg/dL)	Limosis	Postprandial
Normal	70~110	Yes	N/A
Abnormal_high	>110	Yes	N/A
Abnormal_low	50~70	Yes	N/A
Normal_1	120~200	No	1 hour
Abnormal_1	50~120 and >200	No	1 hour
Normal_2	70~140	No	2 hours
Abnormal_2	50~70 and >140	No	2 hours

**Table 2:** Results of the accuracy test.

Accuracy	VFDT	iOVFDT	Bayes	Perceptron
Mean	87.4314%	86.4102%	82.6453%	25.4779%
Max	95.6810%	93.7930%	95.1720%	91.6670%
Min	78.8460%	79.3100%	25.3010%	0.0000%
Std. dev.	0.0403	0.0342	0.1184	0.3164
Quartiles 25	85.4633	84.4560	81.5950	0.0000
Quartiles 50	87.3395	86.1990	85.6170	11.4720
Quartiles 75	90.2530	89.0150	88.4955	53.8140

**Table 3:** The Kappa statistics for the candidate classifiers.

Algorithm	VFDT	iOVFDT	Bayes
Kappa statistics	0.587	0.605	0.678

Reference	Remarks		

0.0~0.20	Slight		
0.21~0.40	Fair		
0.41~0.60	Moderate		
0.61~0.80	Substantial		
0.81~1	Almost perfect		

**Table 4:** Contingency table and the remarks.

	Actual class (observation)	
Predicted class (expectation)	TP (true positive)	FP (false positive)
	Correct result	Unexpected result
	FN (false negative)	TN (true negative)
	Missing result	Correct absence of result

**Table 5:** Contingency table for multiclasses classifiers.

Actual values	Predictions outcomes
$\begin{array}{c} \text{T}{\text{P}}_{1}\\ \;C_{11} \end{array}$	$\begin{array}{cccc} \text{F}{\text{N}}_{1} & & & \\ \;C_{12} & C_{13} & \cdots \; & C_{1d} \end{array}$

$\begin{array}{c} \text{F}{\text{P}}_{\text{1}}\\ \;C_{21}\\ \;\vdots \\ \;C_{d1} \end{array}$	$\begin{array}{cccc} \text{T}{\text{N}}_{\text{1}} & & & \\ \;C_{22} & C_{23} & \cdots & C_{2d}\\ \;\vdots & \vdots & \vdots & \vdots \\ {\;C}_{d2} & C_{d3} & \cdots & C_{dd} \end{array}$

**Table 6:** Numeric results of AUCs of the classifiers.

Test result Variable(s)	Area	Std. error^a^	Asymptotic sig.^b^	Asymptotic 95% confidence interval	
				Lower bound	Upper bound
VFDT	0.752	0.007	0	0.739	0.765
iOVFDT	0.745	0.007	0	0.732	0.758
Bayes	0.847	0.005	0	0.836	0.858
Perceptron	0.508	0.007	0.228	0.495	0.521

The test result variable(s): VFDT, iOVFDT, Bayes, and Perceptron has at least one tie between the positive actual state group and the negative actual state group. Statistics may be biased.

^a^Under the nonparametric assumption.

^b^Null hypothesis: true area = 0.5.

**Table 7:** Numeric results of AUCs of the classifiers.

Classifier	VFDT	iOVFDT	Bayes	Perceptron
Precision	0.894	0.892	0.938*	0.827
Recall	0.991*	0.985	0.953	0.245
*F*-measure	0.940	0.936	0.945*	0.436

*Refers to a winning classifier that has the highest performance.
